# Immunomodulatory Effects of (24R)-Pseudo-Ginsenoside HQ and (24S)-Pseudo-Ginsenoside HQ on Cyclophosphamide-Induced Immunosuppression and Their Anti-Tumor Effects Study

**DOI:** 10.3390/ijms20040836

**Published:** 2019-02-15

**Authors:** Zeng Qi, Lixue Chen, Zhuo Li, Zijun Shao, Yuli Qi, Kun Gao, Songxin Liu, Yinshi Sun, Pingya Li, Jinping Liu

**Affiliations:** 1 School of Pharmaceutical Sciences, Jilin University, Changchun 130021, China; qizeng95@163.com (Z.Q.); zhuoli198602@gmail.com (Z.L.); lipy@jlu.edu.cn (P.L.); 2 Institute of Special Animals and Plants Sciences, Chinese Academy of Agricultural Sciences, Changchun 130112, China; 82101172456@caas.cn (L.C.); shaozijun2017@163.com (Z.S.); 15568871905@163.com (Y.Q.); GaoK2018@163.com (K.G.); liusx2018@163.com (S.L.); 3 College of Chinese Medicinal Materials, Jilin Agricultural University, Changchun 130118, China

**Keywords:** ginsenoside Rh_2_, (24R)-pseudo-ginsenoside HQ, (24S)-pseudo-ginsenoside HQ, immunomodulatory, cyclophosphamide, immunosuppression, anti-tumor activity in vivo

## Abstract

(24R)-pseudo-ginsenoside HQ (R-PHQ) and (24S)-pseudo-ginsenoside HQ (S-PHQ) are the main metabolites of (20S)-ginsenoside Rh_2_ (Rh_2_) in vivo. In this study, we found that Rh_2_, R-PHQ, and S-PHQ upregulated the innate and adaptive immune response in cyclophosphamide (CTX) induced-immunocompromised mice as evidenced by the number of leukocytes, cellular immunity, and phagocytosis of macrophages. Spleen T-lymphocyte subpopulations and the serum cytokines level were also balanced in these immunosuppressed mice. Furthermore, co-administration with R-PHQ or S-PHQ did not compromise the antitumor activity of CTX in the hepatoma H22-bearing mice. Treatment with R-PHQ and S-PHQ clearly induced the apoptosis of tumor cells, significantly increased the expression of Bax, and remarkably inhibited the expression of Bcl-2 and vascular endothelial growth factor (VEGF) in H22 tumor tissues. The anti-tumor activity of R-PHQ and S-PHQ could be related to the promotion of tumor apoptosis and inhibition of angiogenesis and may involve the caspase and VEGF signaling pathways. This study provides a theoretical basis for further study on R-PHQ and S-PHQ.

## 1. Introduction

Cyclophosphamide (CTX) is one of the most widely used antitumor agents in clinical chemotherapy, the application of which often results in leukopenia, immunosuppression, and myelosuppression [1]. Considering that CTX causes abrupt changes to the T helper cells Th1/Th2 bias similar to those seen in tuberculosis, the CTX-induced immunosuppressive mouse model has been used for research on fungal pathogenicity and mechanistic investigations of therapeutics for tuberculosis [2].

Ginsenosides, the main active ingredient in *Panax ginseng, Panax quinquefolium*, and *Panax notoginseng*, are often classified into four groups: Protopanaxadiol type, protopanaxatriol type, oleanane-type, and ocotillol-type [3,4]. Numerous studies have demonstrated that ginsenosides are the major active compounds of antioxidant, anti-obesity, anti-inflammation, neuroprotective, anti-tumor, anti-coagulant, and immunoregulatory effects in *P*. *ginseng* [5,6]. Some ginsenosides, such as 20(R)-ginsenoside Rg_3_ (Shen Yi Capsule, Jilin YATAI Group Pharmaceutical Investment Co., Ltd.), have been clinically applied as tumor neovascularization inhibitors in China [7,8]. However, oral administration of ginsenosides has shown poor absorption and low bioavailability, which has limited their clinical application [9]. To improve the bioavailability and enhance their pharmacologic activities, numerous scientists have pursued chemical structure modifications of ginsenosides [10].

Pharmacokinetic studies revealed oxygenated metabolites to be the major circulating metabolic products of ginsenosides after oral administration [11,12]. The C-24, 25-double bond is the site of oxygenation for the generation of additional hydroxyl or epoxy groups [13]. It was reported that dammarane-type ginsenoside Rg_2_ and ginsenoside Rg_3_ are transformed into corresponding ocotillol-type metabolites of pseudo-ginsenoside F_11_ and pseudo-ginsenoside GQ (PGQ) in artificial gastric juice. These metabolites were confirmed through comparison with synthesized authentic compounds [14]. Thus, we can infer that the monooxygenated metabolite (m2, molecular weight = 638) of Rh_2_, the monooxygenated metabolite (m1, molecular weight = 800) of Rg_3_, and the monooxygenated metabolite (m5, molecular weight = 476) of protopanaxdiol may be the 20, 24-epoxide metabolites, pseudo-ginsenoside HQ (PHQ), PGQ, and pseudosapogenin DQ (PDQ) [11,12]. Such a conclusion indicates that metabolites with 20, 24-epoxy groups are one of the final forms of ginsenosides after oral administration. Hence, it is necessary to synthesize enough ginsenoside metabolites with 20, 24-epoxy groups and to investigate their pharmacological activities. Ocotillol-type saponins were first semi-synthesized by Liu et al. with combinatorial chemistry; PGQ, PHQ, and PDQ were obtained with oxidation cyclization products of ginsenosides from the leaves and stems of *P. quinquefolium* [15]. 

Drug metabolism is the metabolic process of drugs in a living organ, and pharmaceutical drugs may be metabolized by various sequential and chemical processes, which involve both phase I reactions and phase II reactions [16,17]. It was reported that Rh_2_-*O* and d-Rh_2_, the octyl ester derivatives and potential in vivo metabolites of ginsenoside Rh_2_, are beneficial for the immune function of hepatoma H22-bearing mice via the inhibition of spleen and thymus gland atrophy and the enhancement of natural killer (NK) cell activity [9,18]. Additionally, Rh_2_-*O* could induce early lysosomal membrane permeabilization and cell cycle arrest. In addition, ginsenoside compound K, the active form of major ginsenosides deglycosylated by intestinal bacteria after oral administration, was found to selectively accumulate in the liver and transform to mono-octyl ester derivatives. Study on its potential metabolite (m1-*O*) showed that m1-*O* could induce apoptosis of H22 tumor cells and alleviate the atrophy of immune organs compared with the chemotherapy drug, cyclophosphamide [19]. 

It was reported that administration of Rh_2_ could reverse CTX-induced leukopenia [20], but the effect of Rh_2_ on CTX-induced immunosuppression remains unknown. Herein, R-PHQ and S-PHQ were efficiently synthesized in vitro with a high yield via oxidization of Rh_2_ in a method reported in previous reports for in vivo pharmacological study. In this study, the immune-modulating effects of Rh_2_, R-PHQ, and S-PHQ were studied in CTX-induced immunosuppression in BALB/c mice, and the anti-tumor activity of R-PHQ and S-PHQ was investigated in H22 tumor-bearing mice for the first time.

## 2. Results and Discussion

### 2.1. Effect of Rh_2_, R-PHQ, and S-PHQ on Cyclophosphamide-Induced Immunosuppression 

The immune system plays a major role in protecting organisms from infectious disease and metastases through layered defenses with increasing specificity [21]. In this study, two in vivo metabolites of Rh_2_—R-PHQ and S-PHQ—were semi-synthesized and their immunomodulatory effects were investigated in a CTX-induced immunosuppression mouse model. 

#### 2.1.1. Effect of Rh_2_, R-PHQ, and S-PHQ on Body Weight and Immune Organ Indexes

**Figure 1:**
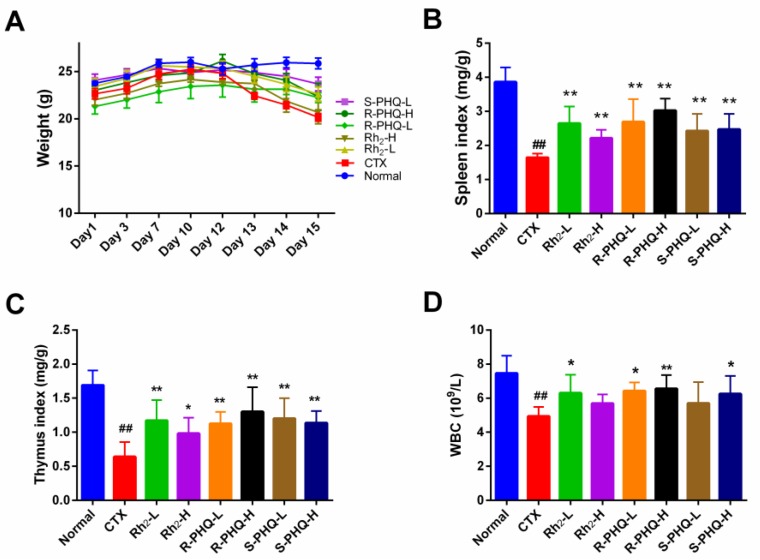
Effect of Rh_2_, R-PHQ, and S-PHQ on (**A**) body weight; (**B**) spleen index; (**C**) thymus index; (**D**) WBC. The values are presented as mean ± SD, *n* = 10. ## *p* < 0.01 compared with the normal group. ** *p* < 0.01 and * *p* < 0.05 compared with the CTX group.

Oral administration of Rh_2_, R-PHQ, and S-PHQ led to no significant changes in body weight, indicating that Rh_2_, R-PHQ, and S-PHQ had no toxic effects on experimental mice (Figure 1A). Body weight, spleen, and thymus indices of the CTX group were significantly decreased compared with the normal group, while the mice treated with Rh_2_, R-PHQ, or S-PHQ showed significantly higher spleen and thymus indices than those of mice in the CTX group (*p* < 0.05) (Figure 1B,C). The spleen index of the R-PHQ-H group was relatively higher than that of the Rh_2_-H and S-PHQ-H group (*p* < 0.05). The above phenomenon manifested that Rh_2_, R-PHQ, and S-PHQ have a positive immunostimulatory effect on the atrophy of immune organs.

#### 2.1.2. Effect of Rh_2_, R-PHQ, and S-PHQ on WBC

Post high-dose CTX chemotherapy for clinical cancer treatment always leads to severe pancytopenia, which could result in potential life-threatening infections [22]. White blood cells (WBC), also called leukocytes, are the cells of the immune system that are involved in protecting the body against both infectious disease and foreign invaders. In our study, after CTX challenge, the peripheral blood WBC in the CTX group significantly decreased as compared with the normal group (*p* < 0.05) (Figure 1D), which indicated a weakened immune system. Pretreatment with Rh_2_, R-PHQ, and S-PHQ induced a good recovery to this change of WBC, which implies that these ginsenosides help to enhance organism immunity.

#### 2.1.3. Effect of Rh_2_, R-PHQ, and S-PHQ on Cellular Immunity

##### Effect of Rh_2_, R-PHQ, and S-PHQ on the Concanavalin A (ConA)-Induced Splenocyte Proliferation

**Figure 2:**
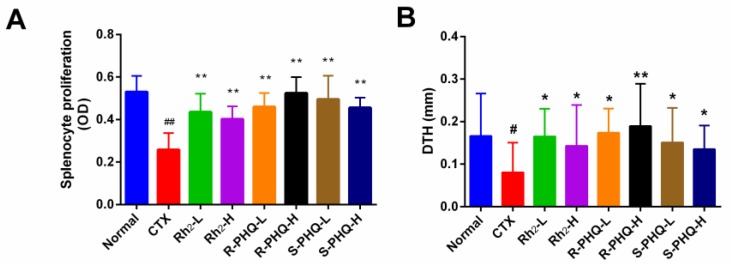
Effect of Rh_2_, R-PHQ, and S-PHQ on the (**A**) ConA-induced splenocyte proliferation and (**B**) SRBC induced DTH. The values are presented as mean ± SD, *n* = 10. ## *p* < 0.01 and # *p* < 0.05 compared with the normal group. ** *p* < 0.01 and * *p* < 0.05 compared with the CTX group.

Lymphocyte proliferation is a pivotal event in the activation cascade of both cellular and humoral immune responses [23]. In this study, we assessed ConA-induced splenocyte proliferation. Compared with the normal group, the CTX group had a significant decrease in splenocyte proliferation (*p* < 0.01). The levels of splenocyte proliferation in mice treated with Rh_2_, R-PHQ, and S-PHQ were significantly higher than the CTX group (*p* < 0.01), which suggested that pretreatment with Rh_2_, R-PHQ, and S-PHQ prevented CTX-induced inhibition of spleen lymphocyte proliferation. Moreover, R-PHQ (20 mg/kg) showed higher splenocyte proliferation than the Rh_2_-H and S-PHQ-H groups (*p* < 0.05) (Figure 2A). 

##### Effect of Rh_2_, R-PHQ, and S-PHQ on Delayed-Type Hypersensitivity (DTH) to Sheep Red Blood Cells (SRBC)

DTH, a type of hypersensitivity reaction, is an important type of cell-mediated pathologic response and plays a pivotal role in the evaluation of the T-cell mediated immune responses [21]. Twenty-four hours later, CTX challenge significantly inhibited DTH to SRBC in the CTX group compared with that in the normal group (Figure 2B). However, we found that Rh_2_, R-PHQ, and S-PHQ attenuated the CTX challenge caused inhibitory effects on the DTH reaction characterized by increasing the footpad thickness of the mouse, suggesting that Rh_2_, R-PHQ, and S-PHQ treatment could descramble the inhibited cell-mediated immune system of CTX-induced immunosuppression mice.

#### 2.1.4. Effect of Rh_2_, R-PHQ, and S-PHQ on Macrophage Phagocytosis

Macrophage is an important part of the innate immune system and plays a critical role in homeostasis. Phagocytosis of pathogens by macrophages initiates the innate immune response, which in turn orchestrates the adaptive response [24]. The phagocytic capacity of macrophage cells and carbon clearance assay are two widely accepted methods for the evaluation of macrophage phagocytosis, thus we applied these two approaches to assess the immunomodulatory effects of ginsenosides.

##### Effect of Rh_2_, R-PHQ, and S-PHQ on the Phagocytic Capacity of Macrophage Cells

**Figure 3:**
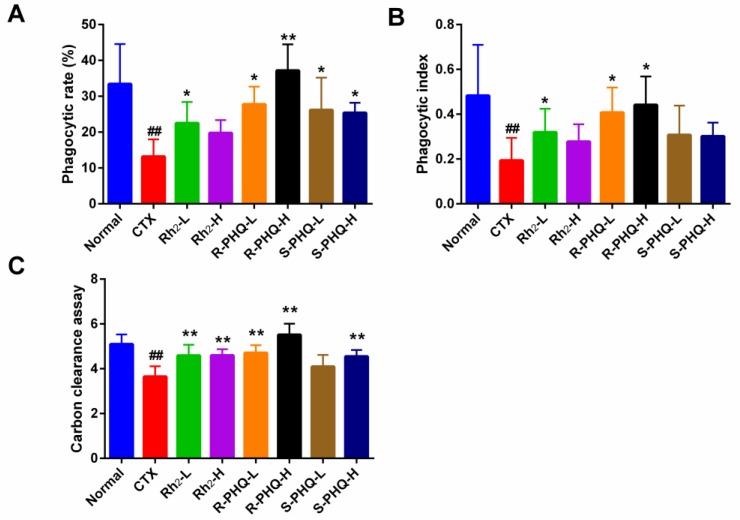
Effect of Rh_2_, R-PHQ, and S-PHQ on the (**A**) phagocytic rate; (**B**) phagocytic index; (**C**) carbon clearance ability. The values are presented as mean ± SD, *n* = 10. ## *p* < 0.01 compared with the normal group. ** *p* < 0.01 and * *p* < 0.05 compared with the CTX group.

According to our phagocytic capacity assay of the macrophage cells, the phagocytic rate and index of the CTX group were markedly decreased compared with the normal group. In varying measures, pretreatment with Rh_2_, R-PHQ, and S-PHQ augmented peritoneal macrophage phagocytosis and promoted the recovery of the phagocytosis ratio of peritoneal macrophage compared with the CTX group (*p* < 0.05) (Figure 3A,B).

##### Effect of Rh_2_, R-PHQ, and S-PHQ on the Carbon Clearance Assay

The carbon clearance assay showed that CTX challenge significantly down-regulated macrophage phagocytosis (*p* < 0.05). The Rh_2_, R-PHQ, and S-PHQ groups showed a higher carbon clearance index as compared with the CTX group (*p* < 0.05) (Figure 3C). The above results provide reliable evidence that Rh_2_, R-PHQ, and S-PHQ could enhance the macrophage phagocytic capacity of CTX-induced immunosuppressed mice.

#### 2.1.5. Effect of Rh_2_, R-PHQ, and S-PHQ on Splenic T-Lymphocyte Subpopulations

**Figure 4:**
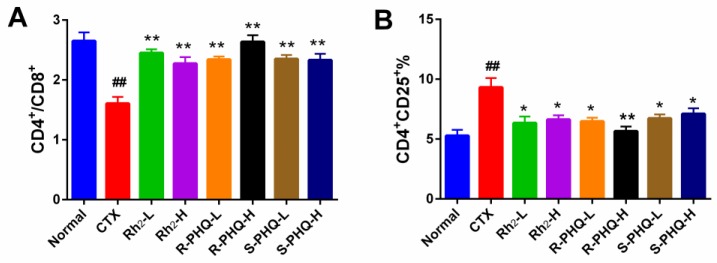
Effect of Rh_2_, R-PHQ, and S-PHQ on the (**A**) CD4+/CD8+; (**B**) CD4^+^ CD25^+^%. The values are presented as mean ± SD, *n* = 10. ## *p* < 0.01 compared with the normal group. ** *p* < 0.01 and * *p* < 0.05 compared with the CTX group.

Splenocytes consist of various immune cells, including T- and B-lymphocytes, macrophages, and dendritic cells. T-cell subpopulations are of great importance in T-cell homeostasis and immune regulation. T-lymphocytes are mainly divided into CD4^+^ T-cells and CD8^+^ T-cells. Helper T-lymphocytes play an important role in enhancing humoral immunity and cellular immunity and can express marker molecule CD4 on their surface. CD8^+^ T-cells, also called killer T-cells, have cytotoxic effects and can specifically kill infected cells and functionally deregulated cells [25]. Compared to the normal group, the ratio of CD4^+^/CD8^+^ was strikingly decreased and CD4^+^ CD25^+^ content was abnormally high in the CTX group (*p* < 0.01). In contrast, pretreatment with Rh_2_, R-PHQ, and S-PHQ significantly recovered the ratio of CD4^+^/CD8^+^ (*p* < 0.01), and markedly inhibited the levels of CD4^+^ CD25^+^ (*p* < 0.05) (Figure 4), indicating that impaired splenic T-lymphocyte sub-populations were recovered by oral administration of Rh_2_, R-PHQ, and S-PHQ.

#### 2.1.6. Effect of Rh_2_, R-PHQ, and S-PHQ on Cytokine Concentrations in Serum

**Figure 5:**
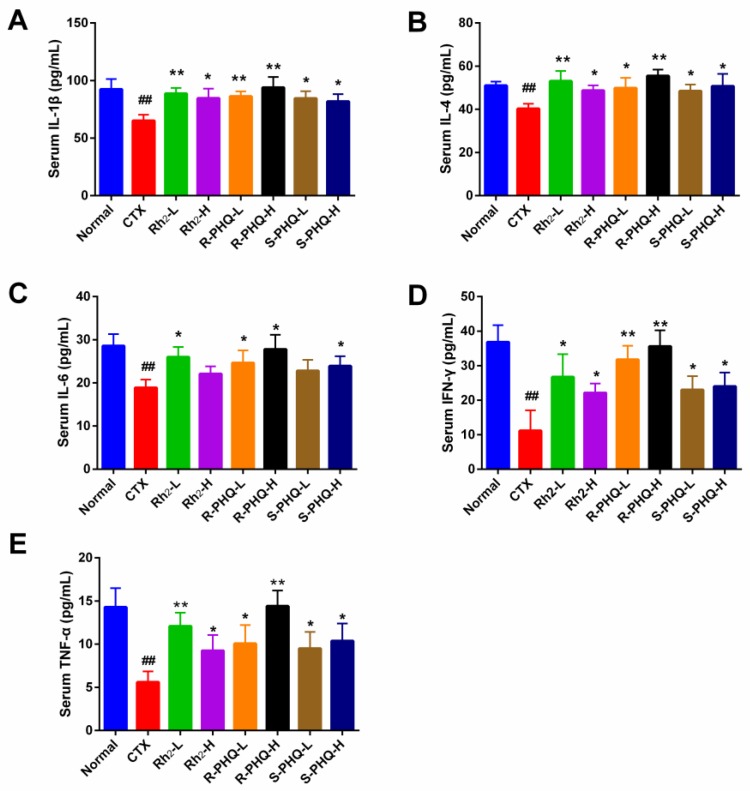
Effect of Rh_2_, R-PHQ, and S-PHQ on the serum levels of (**A**) IL-1β; (**B**) IL-4; (**C**) IL-6; (**D**) IFN-γ; (**E**) TNF-α. The values are presented as mean ± SD, *n* = 8. ## *p* < 0.01 compared with the normal group. ** *p* < 0.01 and * *p* < 0.05 compared with the CTX group.

Cytokines act through receptors and are especially important in the immune system. They modulate the balance between humoral and cell-based immune responses and regulate the maturation, growth, and responsiveness of particular cell populations. Interleukin 1 beta (IL-1β) promotes antimicrobial immunity in macrophages, interferon gamma (IFNγ) and tumor necrosis factor alpha (TNF-α) participate in cell-mediated immune responses, and IL-4 and IL-6 promote humoral or allergic responses [26]. As shown in Figure 5, the levels of IL-1β, IL-4, IL-6, IFN-γ, and TNF-α in serum were significantly decreased in the CTX group compared with the normal group (*p* < 0.01). However, those effects were suppressed by the administration of Rh_2_, R-PHQ, and S-PHQ, which appeared to increase the serum concentration of these cytokines in various degrees (*p* < 0.05). 

Our present experiment confirmed the immuno-regulation activities of Rh_2_ and its derivatives, R-PHQ and S-PHQ, in CTX-induced immunosuppressed mice. Considering CTX is the first-line treatment for cancers, to confirm that co-administration of R-PHQ or S-PHQ with CTX would compromise its antitumor activity or show synergetic effects, H22 hepatocellular carcinoma bearing mice were used to study the anti-tumor activity of R-PHQ and S-PHQ. 

### 2.2. Anti-Tumor Activity

#### 2.2.1. Inhibition Effect of R-PHQ and S-PHQ on H22 Hepatocellular Carcinoma 

The results of the anti-tumor activity of Rh_2_, R-PHQ, and S-PHQ in vivo are summarized in Table 1. Treatment with Rh_2_, R-PHQ, or S-PHQ caused a conspicuous decrease of the tumor weight in the H22-tumor bearing mice as compared with the model control group in a dose-dependent manner. Among these three ginsenosides, R-PHQ exhibited the highest tumor inhibiting rate, and tumor inhibition effect of R-PHQ at the dose of 10 mg/kg was comparable to those observed in the Rh_2_ group at the dose of 20 mg/kg. Additionally, we found that co-administration of R-PHQ with CTX did not compromise its antitumor activity, while co-treatment S-PHQ with CTX showed a synergetic antitumor effect.

#### 2.2.2. Promoting the Apoptotic Effect of R-PHQ and S-PHQ on H22 Hepatocellular Carcinoma

**Figure 6:**
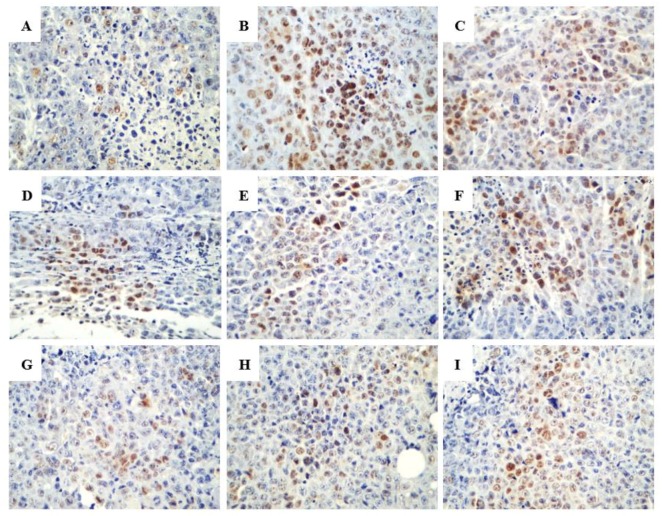
Representative histologic examination of morphological changes in tumors from H22-tumor bearing mice. Tumor sections were analyzed by the TUNEL assay to indicate cell apoptosis (400×). (**A**) model control, (**B**) CTX, (**C**) Rh_2_-H, (**D**) R-PHQ-L, (**E**) R-PHQ-M, (**F**) R-PHQ-H, (**G**) S-PHQ-L, (**H**) S-PHQ-M, (**I**) S-PHQ-H.

**Figure 7:**
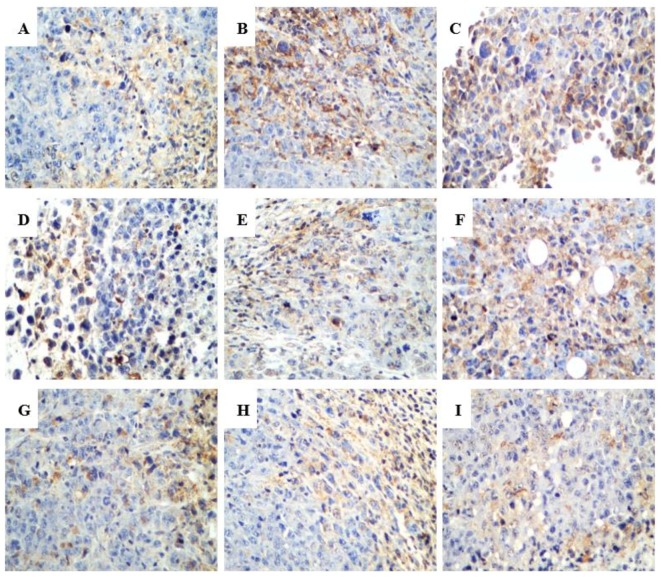
Representative histologic examination of morphological changes in tumors from H22-tumor bearing mice. Protein expression of Bax in H22-tumor tissues was examined by immunohistochemistry (400×). (**A**) model control, (**B**) CTX, (**C**) Rh_2_-H, (**D**) R-PHQ-L, (**E**) R-PHQ-M, (**F**) R-PHQ-H, (**G**) S-PHQ-L, (**H**) S-PHQ-M, (**I**) S-PHQ-H.

**Figure 8:**
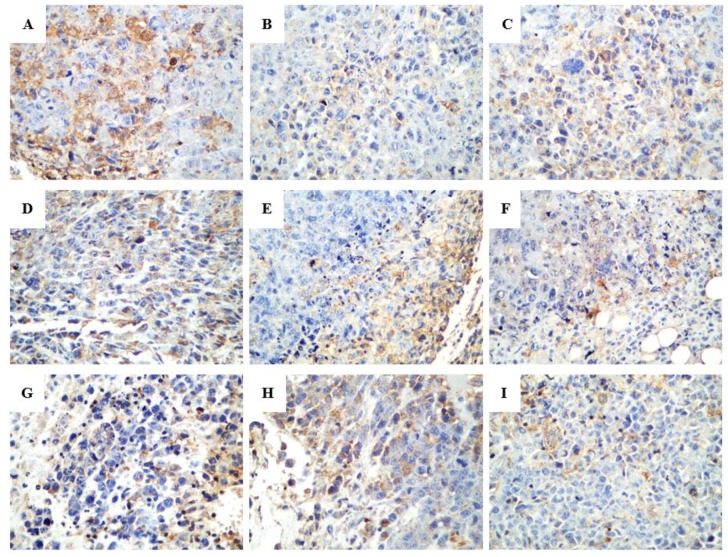
Representative histologic examination of morphological changes in tumors from H22-tumor bearing mice. Protein expression of Bcl-2 in H22-tumor tissues was examined by immunohistochemistry (400×). (**A**) Model control, (**B**) CTX, (**C**) Rh_2_-H, (**D**) R-PHQ-L, (**E**) R-PHQ-M, (**F**) R-PHQ-H, (**G**) S-PHQ-L, (**H**) S-PHQ-M, (**I**) S-PHQ-H.

In the terminal deoxynucleotidyl transferase dUTP nick end labeling (TUNEL) assay, the brown granules were considered as positively stained cells. As shown in Figure 6, drug treatment led to an increased number of cells undergoing apoptosis. Various proteins, such as Fas receptors and caspases, promote apoptosis, while some proteins in the Bcl-2 family, including Bcl-2, inhibit apoptosis. Bax is a pro-apoptotic protein that is expressed in the cytoplasm. As shown in Figure 7 and Figure 8, both R-PHQ and S-PHQ caused clearly higher Bax expression and lower Bcl-2 expression than that in the model control group. 

**Figure 9:**
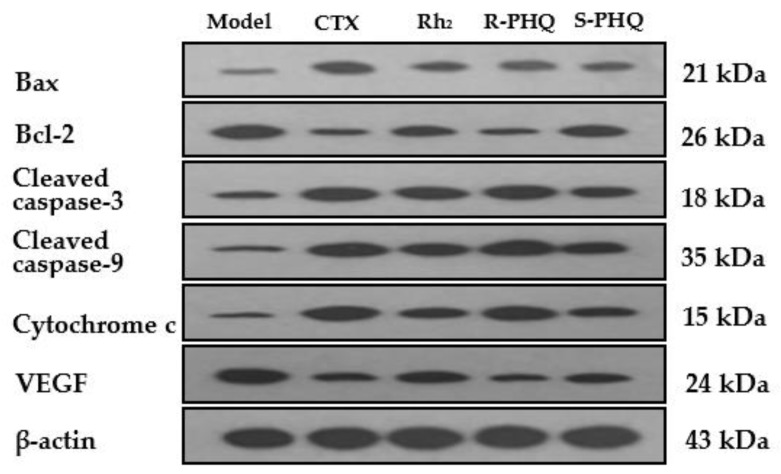
Effects of R-PHQ (20 mg/kg) and S-PHQ (20 mg/kg) on the protein expression of Bax, Bcl-2, caspase-3, caspase-9, cytochrome c, and VEGF were examined by Western blot (of three independent experiments performed).

In addition, Western blot showed that Rh_2_, R-PHQ, and S-PHQ significantly upregulated the protein expression of Bax, cytochrome c, caspase-3, and caspase-9, and suppressed the level of anti-apoptotic protein Bcl-2 in various degrees (Figure 9).

#### 2.2.3. Effect of R-PHQ and S-PHQ on Tumor Angiogenesis 

**Figure 10:**
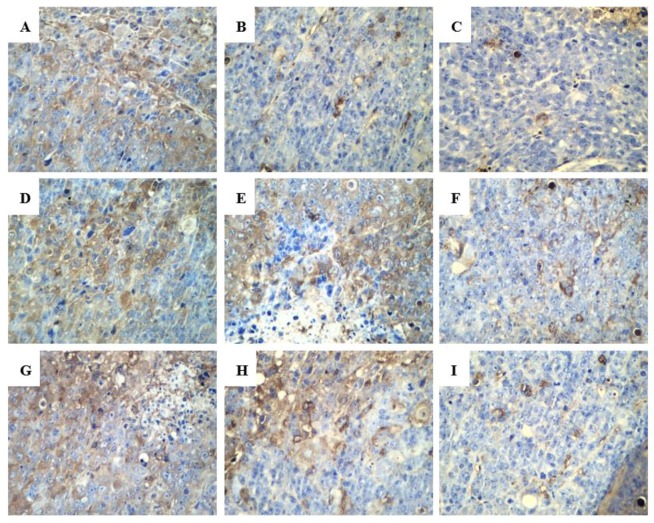
Representative histologic examination of morphological changes in tumors from H22-tumor bearing mice. Protein expression of VEGF in H22-tumor tissues was examined by immunohistochemistry (400×). (**A**) Model control, (**B**) CTX, (**C**) Rh_2_-H, (**D**) R-PHQ-L, (**E**) R-PHQ-M, (**F**) R-PHQ-H, (**G**) S-PHQ-L, (**H**) S-PHQ-M, (**I**) S-PHQ-H.

Vascular endothelial growth factor (VEGF) is an important signal protein produced by cells that stimulate the formation of blood vessels [27]. Photomicrograph results showed that VEGF expression was very high in the model control group (Figure 10). However, treatment with R-PHQ and S-PHQ dose-dependently reduced the positive expression of VEGF. A similar phenomenon was observed in Figure 9, as compared with the model control group, R-PHQ and S-PHQ remarkably inhibited the expression of VEGF, which illustrates that the antitumor activity of R-PHQ and S-PHQ may be relevant to the inhibition of angiogenesis.

Hepatocellular carcinoma (HCC) is one of the most common malignant tumors around the world, characterized by poor prognosis and high mortality [28]. H22 tumor is widely used in the xenograft mouse model for the evaluation of the growth-inhibiting effect of anti-cancer lead compounds in vivo [29] Here, we demonstrated that R-PHQ and S-PHQ significantly suppressed the growth of H22 tumor in a dose-dependent manner. H22 tumor is a hypervascular tumor, and its growth and metastasis are partly dependent on angiogenesis [30]. VEGF, vascular endothelial growth factor, is an important signal protein and marker produced by cancer cells that stimulates angiogenesis [31]. Immunohistochemistry (IHC) analysis showed strong VEGF positive expression in the H22 tumor tissue. However, treatment with R-PHQ and S-PHQ inhibited the expression of VEGF in a dose-dependent manner, coinciding with the decreased VEGF levels after drug intervention in the Western blot analysis. 

Under normal circumstances, apoptosis acts as the fundamental role for the physiological balance in multicellular organisms [3]. Recently, emerging reports demonstrated that anticancer effects of natural products and their derivatives were involved in the pro-apoptotic action, which is considered as a promising approach for the treatment of malignancy [32,33]. Previous studies have reported that Rh_2_ could induce human hepatoma SK-HEP-1 cells apoptosis via Bax/Bak triggered cytochrome c release and caspase-9/caspase-8 activation to execute cancer cell death [34]. In this study, apoptotic cells were checked by the TUNEL assay in the tumor sections from H22 tumor-bearing mice. Both R-PHQ and S-PHQ treatment groups induced cell apoptosis in the transplanted H22 tumor. Interestingly, our results also showed that R-PHQ and S-PHQ could regulate the levels of apoptosis-related proteins. The release of cytochrome c from the mitochondrial intermembrane to the cytosol was proven as a key factor in the activation of caspase-9, which subsequently initiates a caspase cascade involving caspase-3 [35,36]. Pro-apoptotic protein, Bax, a Bcl-2 family member, is responsible for the triggering of cytochrome c release and the induction of apoptosis [37], while the anti-apoptotic protein, Bcl-2, exerts an inhibiting effect on the function of Bax and is known as a cause of resistance to chemotherapeutic drugs in cancer treatments [38]. The IHC analyses of tumor tissues showed that after R-PHQ or S-PHQ treatment, the expression of Bax was markedly up-regulated while the level of Bcl-2 was significantly reduced. In addition, these results were verified by Western blot. Immunoblotting demonstrated that R-PHQ or S-PHQ administration led to the enhanced expression of apoptosis-promoting proteins, Bax, cytochrome c, caspase-3, and caspase-9, while the level of the anti-apoptotic protein, Bcl-2, was inhibited in the H22 tumor tissues. This close association of cytochrome c release with subsequent activation of caspase 9 and caspase 3 suggested that R-PHQ and S-PHQ could induce the apoptosis of hepatoma H22 cells through the mitochondrial pathway.

Our findings corroborate the results of previous studies in which Rh_2_ and its octyl ester derivatives, Rh_2_-O and D-Rh_2_, helped hepatoma H22-bearing mice modulate their immune response. Specifically, the response was characterized by the inhibition of thymus gland atrophy as well as spleen and the thymus indices, as well as increasing the percentages of CD3^+^ T lymphocyte, NK cells activity, the ratio of CD4^+^/CD8^+^ subgroups, and serum IL-2 and TNF-*α* production [18]. Stereo-configuration is critical to the bioactivity of ginsenosides, with even small differences in the configuration of chemical structures leading to sharp differences in bioactivities. Comparative study of 20(R)-Rh_2_ and 20(S)-Rh_2_ showed that the 20(S)-Rh_2_ form exhibited more evident antic2ancer activity through the suppression of cell proliferation [39]. Stereospecificity of ginsenoside Rg_3_ has a great influence on the antitumor effect and promotion of cellular immunity in H22-tumor-bearing mice. The in vivo efficacy study showed that the rate of inhibition of tumor growth for 20(R)-Rg_3_ (40.9%) was considerably higher than 20(S)-Rg_3_ (23.6%), and the cellular immunity regulating effects of 20(R)-Rg_3_ were significantly greater than those of the S-form [40]. In our study, both R-PHQ and S-PHQ showed fine immunomodulatory activity in CTX-induced immunosuppressed mice. Additionally, R-PHQ exhibited a relatively better antitumor effect than its C-24 stereoisomer, S-PHQ, in H22 tumor-bearing mice. At the same time, we found an interesting phenomenon that S-PHQ showed a synergetic antitumor effect with CTX in the co-treatment experiment. The reason for the differences in the reaction between R-PHQ and S-PHQ treatment as detected in our study is currently unknown, so further investigation is warranted. 

## 3. Materials and Methods

### 3.1. Reagents and Animals

Rh_2_, R-PHQ, and S-PHQ were prepared in our lab, Jilin University School of Pharmaceutical Sciences (preparation method and structure information included in the supporting file). RPMI-1640 medium, fetal bovine serum (FBS), benzylpenicillin, and streptomycin were offered by Gibco (Grand Island, NY, USA). Dimethyl sulfoxide, ConA, and MTT were provided by Sigma-Aldrich Co., (St Louis, MO, USA). Cyclophosphamide was purchased from Shengdi Pharmaceutical Co., Ltd. (Jiangsu, China). The antibodies for the T-cell subpopulations assay, including fluorescein isothiocyanate (FITC)-conjugated rat anti-mouse CD4, allophycocyanin (APC)-conjugated rat anti-mouse CD8a, and phycoerythrin (PE)-conjugated rat anti-mouse CD25, were obtained from BioLegend (San Diego, CA, USA). 

BALB/c mice and ICR mice (male, 18–22 g) were provided by Changsheng Biotechnology Co., Ltd. (Liaoning, China). Mice were raised in a standard lab environment (12 h light/dark cycle, 23 ± 1 °C, relative humidity: 50 ± 5%) with free access to food and water. Subjects were fasted 12 h prior to the experimentation with free access to water only. All experiments were executed strictly according with the Principle of Laboratory Animal Care and the guidelines prescribed by the Animal Research Committee of the Institute of Special Animals and Plants Sciences, Chinese Academy of Agricultural Sciences (Permit No.: ECLA-ISAP-18079; date of approval: 5 October 2018). 

### 3.2. Establishment of Cyclophosphamide-Induced Immunosuppression Experimental Animal Model and Drugs Evaluation 

After one-week acclimatization, BALB/c mice were randomly divided into eight groups (*n* = 10): (1) Normal, (2) CTX (75 mg/kg), (3) CTX + Rh_2_-L (10 mg/kg), (4) CTX + Rh_2_-H (20 mg/kg), (5) CTX + R-PHQ-L (10 mg/kg), (6) CTX + R-PHQ-H (20 mg/kg), (7) CTX + S-PHQ-L (10 mg/kg), (8) CTX + S-PHQ-H (20 mg/kg) [9,18,41]. Rh_2_, R-PHQ and S-PHQ were suspended in 0.05% carboxymethylcellulose sodium (CMC-Na) and orally administered to all mice for 15 days. Mice in the normal and CTX groups were treated with 0.05% CMC-Na. The CTX group was subjected to intraperitoneal injection of CTX at a dosage of 75 mg/kg on day 12–14. Blood samples from the ophthalmic venous plexus were then collected. Finally, the spleens and thymuses were rapidly isolated and weighed, of which the indices (organ weight—body weight ratio) were calculated.

#### 3.2.1. SRBC-Induced DTH

One hour after the last injection of CTX, the mice were intraperitoneally injected with 2% defibrinated SRBCs for the DTH assay. On day 15, a vernier caliper was used to measure the baseline footpad thickness of the left rear foot of all mice. Subsequently, each mouse was given a subcutaneously injection of 20 μL of 20% (*v*/*v*) SRBCs (1 × 10^8^ cell) into the left rear footpad, meanwhile the thickness of the rear footpads was measured after 24 h. The mean value of the measuring results of three measurements was used as the final result. The difference of the footpad thickness indicated the effect of ginseng on cellular immunity.

#### 3.2.2. Carbon Granular Clearance assay

On day 16, mice were injected intravenously into the coccygeal vein with 0.1 mL/10 g of India ink that was diluted 4 times with sterile saline. Twenty microliters of blood samples were collected from the ophthalmic venous plexus at 2 and 10 min after the injection. Two mL 0.1% sodium carbonate solution was mixed with the blood sample. The OD values were measured at 600 nm by a Microplate Reader (BioTek Instruments, Inc., Winooski, VT, USA). After blood sampling, the animals were euthanized and the spleens and livers were isolated and weighed. The phagocytic index, *α*, was applied to validate the function of the mouse phagocytes in clearing carbon particles and calculated using the following equations: *α* = body weight × $\sqrt[{3}]{k}/$(liver weight + spleen weight); *k* = (lg OD_1_ − lg OD_2_)/(T_2_ − T_1_).

#### 3.2.3. Phagocytic Function of Peritoneal Macrophage

On day 16, the mice were immunized by intraperitoneal injection of 1 mL of 20% chicken red blood cells (CRBCs) and then sacrificed 30 min after the injection. Peritoneal cells were obtained from the peritoneal cavity using a peritoneal lavage, and then suspended in 2 mL of saline solution. Aspirating 1mL of cell-rich lavage fluid was sucked and smeared on glass slides before being incubated at 37 °C for 30 min. Non-adherent cells were washed off with saline solution, whereas the macrophage cells were fixed with an acetone-methanol mixture (1:1, *v*/*v*) before being stained with 4% Giemsa-PBS solution. After that, the stained cells were subjected to distilled water rinsing and air-drying before being counted with an inverted microscope (Leica Microsystems, Wetzlar, Germany) at 40× magnification for the calculation of the phagocytic rate and phagocytic index. The phagocytic rate was the percentage of macrophages that phagocytosed CRBCs per 100 macrophages. The phagocytic index was the number of CRBCs that were phagocytosed per 100 macrophages.

#### 3.2.4. Splenocyte Proliferation Assay

On day 16, the spleen was collected from the sacrificed mice under aseptic conditions. Then, erythrocytes debris and clumps were removed to obtain a single cell suspension. Splenocytes were washed three times with PBS and then suspended in a solution of complete RPMI-1640 medium with a final density of 3 × 10^6^ cells/mL. Spleen cells were cultivated into 24-well plates without (control wells) or with 75 μL of ConA as a T cell stimulant, and then incubated at 37 °C under humid 5% CO_2_ conditions for 64 h. After incubation, 0.7 mL of medium was discharged from each well, and then 0.7 mL of RPMI-1640 without FBS and 50 μL of 5 mg/mL MTT were added for the replacement. After incubation at 37 °C and 5% CO_2_ for 4 h, 1 mL of acidic isopropanol solution was added to each well to dissolve the insoluble purple formazan product. Subsequently, the OD values were measured at 570 nm with a Microplate Reader (BioTek Instruments, Inc., Winooski, VT, USA). Each measurement was performed 3 times. After being treated with ConA, the proliferation capacity was characterized by the difference in absorbance.

#### 3.2.5. Mouse Leukocyte Assay and Splenic T-Lymphocyte Subpopulations Assay

On day 16, a 20 μL blood sample was collected from the ophthalmic venous plexus and diluted in 0.38 mL Turk’s solution. Then, the leucocytes were counted by a microscope.

For the splenic T-lymphocyte subpopulations assay, the splenocyte suspension was adjusted to 1 × 10^6^ cells/mL, and subjected to flow cytometry to measure the splenocyte lymphocyte subpopulations. The splenocyte surface markers were labeled with fluorescein isothiocyanate (FITC)-conjugated anti-mouse CD4, APC-conjugated anti-mouse CD8a, and PE-conjugated anti-mouse CD25. The labeled cells were washed twice, resuspended in staining buffer (BioLegend), and analyzed using a FACSCalibur (BD Medical Technologymanufacturer, Franklin Lakes, NJ, USA) and CellQuest software. As a comparison, the cells stained with isotype-matched antibodies were adopted to calibrate the FACSCalibur instrument settings.

#### 3.2.6. Determination of Cytokines in Serum

The blood samples were centrifuged at 3000 rpm for 10 min, and then the serum was stored at −80 °C. The experiment was carried out by the enzyme-linked immunosorbent assay (ELISA) double antibody sandwich method using the ELISA Kit (Invitrogen Co., Ltd., Carlsbad, CA, USA).

### 3.3. Establishment of the Xenograft Tumor Model and Drug Treatment

The mouse H22-hepatoma cell line was obtained from Shanghai Institute of Biochemistry and Cell Biology, China. Murine H22 cells from the ascitic fluid were diluted with PBS, and 0.2 mL tumor cell suspension (1 × 10^5^ cells/mL) was subcutaneously injected into the right forelimb armpit for each ICR mouse. After 24 h, these mice were randomly divided into 13 groups (*n* = 8): (1) Model control, (2) CTX (25 mg/kg), (3) Rh_2_-L (5 mg/kg), (4) Rh_2_-M (10 mg/kg), (5) Rh_2_-H (20 mg/kg), (6) R-PHQ-L (5 mg/kg), (7) R-PHQ-M (10 mg/kg), (8) R-PHQ-H (20 mg/kg), (9) S-PHQ-L (5 mg/kg), (10) S-PHQ-M (10 mg/kg), (11) S-PHQ-H (20 mg/kg), (12) CTX (25 mg/kg) + R-PHQ-H (20 mg/kg), (13) CTX (25 mg/kg) + S-PHQ-H (20 mg/kg). The CTX group was treated with an intraperitoneal injection. The mice were injected with CTX, or orally administered with Rh_2_, R-PHQ, or S-PHQ 1 time/d for 14 days. Mice in the model control group were treated with 0.05% CMC-Na. Finally, blood was collected from the orbit, and they were sacrificed by cervical dislocation, and tumors were separated and weighted. TUNEL assay, immunohistochemistry analysis, and Western blotting were performed as previously described [42]. Western blotting was used to analyze the levels of anti-apoptotic factor, Bcl-2; the pro-apoptotic factors, BAX, cytochrome c, caspase-3, and caspase-9; and VEGF.

### 3.4. Statistical Analysis

Data were analyzed with GraphPad Prism 6.0 software (GraphPad Software Inc., San Diego, CA, USA) and presented as mean ± S.D. Statistical significance was calculated with a two tailed test or a one-way analysis of variance (ANOVA) and *p*-value < 0.05 was considered as significant.

## 4. Conclusions

In summary, our present experiment reported the immuno-regulation activities of Rh_2_ and its derivatives, R-PHQ and S-PHQ, in CTX-induced immunosuppressed mice. Importantly, we found R-PHQ and S-PHQ, the 20, 24-epoxide derivatives of Rh_2_, did not compromise the antitumor activity of CTX in H22 tumor-bearing mice. The present study provides a lead for the research of the synthetic study of R-PHQ and S-PHQ as well as other Rh_2_ derivatives.

## Figures and Tables

**Table 1:** Tumor weight and inhibition rate of tumor growth on H22 tumor-bearing mice.

Groups	TW (g)	TIR (%)
Model control	2.46 ± 0.66	-
CTX	1.06 ± 0.20 **	57.09%
Rh_2_ (5 mg/kg)	2.09 ± 0.41	15.12%
Rh_2_ (10 mg/kg)	1.65 ± 0.32 **	32.91%
Rh_2_ (20 mg/kg)	1.28 ± 0.30 **	47.83%
R-PHQ (5 mg/kg)	1.54 ± 0.33 **	37.34%
R-PHQ (10 mg/kg)	1.32 ± 0.49 **	46.24%
R-PHQ (20 mg/kg)	1.27 ± 0.30 **	48.52%
S-PHQ (5 mg/kg)	1.94 ± 0.50 **	21.25%
S-PHQ (10 mg/kg)	1.65 ± 0.54 **	32.83%
S-PHQ (20 mg/kg)	1.35 ± 0.38 **	44.98%
CTX + R-PHQ (20 mg/kg)	0.99 ± 0.37 **	59.88%
CTX + S-PHQ (20 mg/kg)	0.80 ± 0.47 **	67.57%

*n* = 10, ** *p* < 0.01 vs. model control group.

## Abbreviations

R-PHQ	(24R)-pseudo-ginsenoside HQ
S-PHQ	(24S)-pseudo-ginsenoside HQ
Rh_2_	(20S)-ginsenoside Rh_2_
CTX	cyclophosphamide
PGQ	pseudo-ginsenoside GQ
PDQ	pseudosapogenin DQ
PHQ	pseudo-ginsenoside HQ
WBC	White blood cells
ConA	Concanavalin A
DTH	Delayed-type hypersensitivity
SRBC	sheep red blood cells
VEGF	Vascular endothelial growth factor
